# Research Progress on Router Devices for the OAM Optical Communication

**DOI:** 10.3390/s24030944

**Published:** 2024-02-01

**Authors:** Binbin Wang, Xizheng Zhang, Jinlong Tian, Badreddine Merabet, Zhixiang Li, Syed Afaq Ali Shah, Yi Lei, Bingyi Liu, Kai Guo, Zhongyi Guo

**Affiliations:** 1 School of Computer and Information, Hefei University of Technology, Hefei 230009, China; wangbinbin2021s@163.com (B.W.); tianjinlong2022s@163.com (J.T.); merabet.badreddine@yahoo.com (B.M.); lizhixiang2021s@163.com (Z.L.); leiyi@hfut.edu.cn (Y.L.); bingyiliu@hfut.edu.cn (B.L.); kai.guo@hfut.edu.cn (K.G.); 2 School of Mechanical Engineering, Hefei University of Technology, Hefei 230009, China; 3 School of Materials Science and Engineering, Hefei University of Technology, Hefei 230009, China; zhangxizheng2021b@163.com

**Keywords:** orbital angular momentum (OAM), OAM router devices (OAM-RDs), channel multicasting, channel switching, channel filtering

## Abstract

Vortex beams carrying orbital angular momentum (OAM) provide a new degree of freedom for light waves in addition to the traditional degrees of freedom, such as intensity, phase, frequency, time, and polarization. Due to the theoretically unlimited orthogonal states, the physical dimension of OAM is capable of addressing the problem of low information capacity. With the advancement of the OAM optical communication technology, OAM router devices (OAM-RDs) have played a key role in significantly improving the flexibility and practicability of communication systems. In this review, major breakthroughs in the OAM-RDs are summarized, and the latest technological standing is examined. Additionally, a detailed account of the recent works published on techniques related to the OAM-RDs has been categorized into five areas: channel multicasting, channel switching, channel filtering, channel hopping, and channel adding/extracting. Meanwhile, the principles, research methods, advantages, and disadvantages are discussed and summarized in depth while analyzing the future development trends and prospects of the OAM-RDs.

## 1. Introduction

In recent years, with the continuous development of the modern information industry and the rapid growth of large traffic consumable businesses, such as the Internet of Things, cloud computing, e-commerce, and online gaming, there has been an increasing demand for high-speed and large-capacity communication among people. This trend is expected to pose a significant challenge to existing communication technologies [1,2,3,4,5]. In the field of free-space optical communication, multi-dimensional multiplexing technologies, such as wavelength-division multiplexing (WDM), polarization-division multiplexing (PDM), and time-division multiplexing (TDM), are commonly employed to enhance system capacity, along with advanced signal modulation techniques, such as quadrature amplitude modulation (QAM), pulse position modulation (PPM), and multi-phase shift keying [6,7,8,9,10]. However, in recent years, the development of these resource dimensions has nearly reached its limits. Network bandwidth has gradually become a bottleneck, and capacity crisis remains a significant challenge for optical communication. Therefore, expanding the communication system’s capacity has become an urgent problem to be addressed in the field of communication [11]. At present, a novel research concept involves utilizing spatial dimensions for multiplexing. Among these dimensions, orbital angular momentum (OAM) stands out as a distinct degree of freedom from polarization, amplitude, phase, frequency, and time due to its exceptional advantages in terms of transmission speed, capacity, and stability. As such, OAM has become the primary focus of current research [12,13,14,15,16,17].

In 1992, Allen et al. [18] experimentally verified that a beam with a helical phase wavefront carries the OAM. The most common type of OAM beam in optical communication systems, the Laguerre–Gaussian (LG) beam’s field distribution, can be mathematically expressed as follows [19]:

(1) $$\begin{array}{ll} u_{LG(l,p)}\left(r,\varphi ,z\right) & =\sqrt{\frac{2p!}{\left(\pi \left(p+\left|l\right|\right)!\right)}}\frac{1}{w\left(z\right)}{\left(\frac{r\sqrt{2}}{w\left(z\right)}\right)}^{\left|l\right|}L_{p}^{\left|l\right|}\left(\frac{2r^{2}}{w^{2}\left(z\right)}\right)\\ & \times \exp\left(\frac{-r^{2}}{w^{2}\left(z\right)}-\frac{ikr^{2}z}{2R\left(z\right)}\right)\\ & \times \exp\left[i\left(2p+\left|l\right|+1\right)\tan^{-1}\frac{z}{z_{R}}\right]\exp\left(il\varphi \right) \end{array}$$

where *l* is referred to as the topological charge or OAM mode value, indicating the number of wavefront rotations within one wavelength propagation distance; *p* is the radial indices; λ is the wavelength; k=2π/λ is the wavenumber; $w(z)=w_{0}\sqrt{1+(z/z_{R}{)}^{2}}$ represents the Gaussian beam radius, in which $w_{0}$ is the beam waist and ZR=πw02λ is the Rayleigh range; $L_{p}^{\left|l\right|}(\cdot )$ represents the generalized Laguerre polynomial; and $\left(2p+\left|l\right|+1)\tan^{-1}(z/z_{R}\right)$ is the Gouy phase. Due to the existence of a phase singularity in the center of the beam, the distribution of the vortex annular field intensity is 0. This type of beam is commonly referred to as an OAM beam or vortex beam [19,20,21,22,23]. The helical phase structure characteristics of a vortex beam are determined by the phase factor exp(*ilφ*), [24,25,26]. *φ* represents azimuths, with each photon in a vortex beam carrying an OAM of *lћ* (where *ћ* is Planck’s constant divided by 2π) [27,28,29]. Theoretically, any value of *l* can be selected, and OAM beams carrying different topological charges are mutually orthogonal [30,31,32]. These exceptional qualities of OAM have guaranteed promising results in addressing the problem of low information capacity in optical communications. With the advancement of OAM optical communication technology, OAM router devices (OAM-RDs) have played a key role in significantly improving the flexibility and practicability of communication systems. Therefore, the multiplexing of OAM beams with different topological charges without interference is possible, which provides a potential solution to the capacity crisis in optical communication. Currently, the OAM beam exhibits a vast range of potential applications in the fields of optics [33,34,35,36,37,38,39,40], electromagnetism [41,42,43,44,45,46,47,48,49,50], acoustics [51,52,53,54,55,56,57,58,59,60], and mechanics [61,62,63], thus garnishing significant attention from researchers.

In the field of optical communication, the applications of OAM beams mainly include two areas: OAM-shift keying (OAM-SK) [64,65,66,67,68,69,70,71] and OAM-division multiplexing (OAM-DM) [72,73,74,75,76,77,78,79]. OAM-SK is a mapping technique that assigns digital signals to differentiate OAM beams based on the unique value of each OAM mode (with each mode representing a data bit). Meanwhile, OAM-DM utilizes the carrier of an OAM beam to modulate signals and achieve channel multiplexing via the orthogonality between various beams, resulting in exponential increases in the channel capacity [80,81,82]. For a mature OAM optical communication system, the manipulation and transformation of different channels play a crucial role in enhancing the flexibility and practicality of the system [83,84,85]. Currently, the research focus in OAM optical communication lies in the development of routing technology that enables end-to-end signal transmission path transformations [86,87,88,89,90,91]. Therefore, extensive research and development have been conducted on the OAM router devices (OAM-RDs) with significant performance enhancements [92,93,94].

This review focuses on the field of OAM-RDs and provides a detailed account of recent advancements in OAM router schemes, which are centered around OAM-RDs that enable channel multicasting, channel switching, channel filtering, channel hopping, and channel adding/extracting functionalities, as illustrated in Table 1. Finally, this study also summarizes the research process of OAM-RDs while examining their potential for future development.

## 2. OAM Channel Multicasting

**Figure 1:**
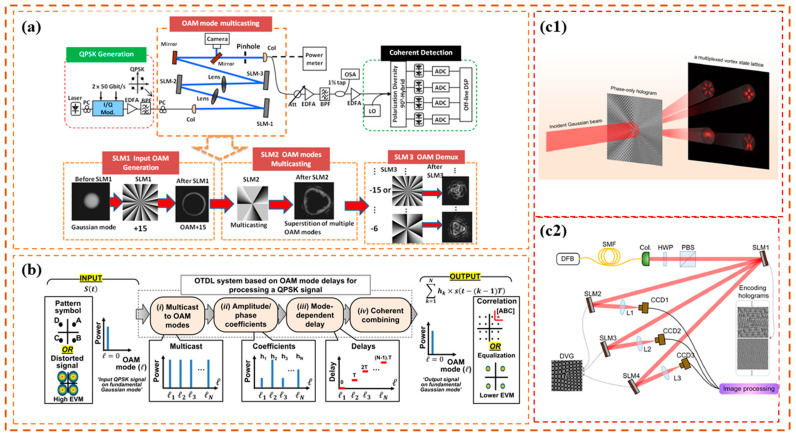
Principle or experimental setup of OAM-RDs based on OAM channel multicasting (I); (**a**) experimental setup for OAM-mode multicasting [104], Col: Collimator; (**b**) the concept of tunable OTDL system using the modal-dependent delays [107]; (**c1**,**c2**) the multiplexed vortex state array [108]: (**c1**) depicts the concept of generating a multiplexed vortex state array using a phase-only hologram, and (**c2**) shows the experimental setup, BS, and beam splitter; L1~L4, lenses; CCD1&CCD2, infrared CCD camera; AS, aperture stop.

In OAM-based optical communication networks, the transmission efficiency of OAM communication links can be greatly expanded by the selective manipulation of different data channels. One such OAM router device (OAM-RD) realizes OAM multicasting by dispersing a single channel into multiple channels and then transmitting data information from a single channel to multiple channels. These channels are orthogonal to each other and can serve different users in multi-user communication so as to better adapt to the multi-user environment. At present, multicast technology is widely used in traditional optical communication and OAM optical communication [95,96,97,98,99,100,101,102]. For an OAM optical communication system, the OAM-RD, which can realize the OAM multicasting, plays an important role [103]. In 2012, Yan et al. proposed a method of spatial mode multicasting in OAM mode spatial division multiplexing [104]. As depicted in Figure 1a, a quadrature-phase-shift-keyed (QPSK) optical signal is transmitted from a single-mode fiber to a fiber collimator. During the OAM mode multicasting stage, when an OAM beam with input topological charge l encounters an angular-amplitude aperture with n-order rotational symmetry, it can be assigned to a multi-OAM beam with equally spaced OAM topological charges [105]. For coherent detection, one of the multicast channels is demultiplexed and then coupled to a single-mode fiber. First, the Gaussian beam emitted from the collimator is transformed into an OAM beam using a spatial light modulator (SLM) loaded with a helical phase distribution to generate a single OAM channel. And then, the input single OAM channel is converted into a superposition of multiple OAM channels using a specially designed slicing phase pattern on SLM-2. SLM-3 is utilized for demultiplexing one of the multicasting channels. After demultiplexing, the beam passes through a pinhole and then couples into a single-mode fiber for coherent detection. Subsequently, this research group demonstrated through experiments that their proposed method can produce up to eight multicast modes with uniform power and less than −20 dB crosstalk, and the power loss induced by the multicasting is only 0.3 dB, as shown in Table 2. The significant advantage of this scheme is that it can generate the required OAM modes while maintaining a low power loss. However, multiple optical devices are needed in the experimental process, and the device integration is relatively low.

In real-world applications, beam power requirements may vary for different users. To address this issue and to improve the overall transmission efficiency, in 2015, Li et al. [106] proposed a multicast scheme with adjustable power by employing a specially designed complex phase pattern to enable the generation of composite beams with multiple OAM modes superimposed with adjustable success rates. In 2022, Almaiman et al. [107] presented a novel approach to creating tunable optical tapped-delay-lines (OTDLs) based on the OAM modal domain. This method leverages OAM mode multicasting to generate the necessary taps, thereby enabling flexible and efficient tunability. Figure 1b illustrates the fundamental concept behind the tunable OTDL, which relies on mode-dependent delays to perform the functions of correlation and equalization on a QPSK data signal. Specifically, the input optical beam carrying a QPSK data signal s(t) in the fundamental Gaussian mode is fed into the OTDL system. The input signal in the tunable OTDL system can consist of either pattern symbols [A, B, C, D] or a QPSK signal constellation that is distorted by chromatic dispersion. The OTDL adopts a multi-step approach: first, the system creates the input signal taps by multicasting into different OAM modes (*l*_1_, *l*_2_, *…*, *l_N_*), where *N* ∈ [1, 2, …, ∞). Second, each of the replicas in the OAM domain is an assigned amplitude and phase coefficient corresponding to a specific tap (*h*1, *h*2, …, *hN*). Finally, the taps are differentially delayed using a mode-dependent delay subsystem (0, *T*, …, (*N* − 1) *T*), allowing for precise temporal control of the signal, and the taps are combined to provide the output $\sum_{k=1}^{N}h_{k}\times s\left(t-\left(k-1\right)T\right)$ in the fundamental mode. Subsequent experiments were conducted to validate the equalization function of the proposed system using a 20 Gbaud QPSK signal as input and subjecting it to 336–1008 ps/nm of chromatic dispersion. The results demonstrate that the system can significantly improve the signal quality, reducing the error vector amplitude (EVM) from 25.4% to 12.9% in the presence of 336 ps/nm chromatic dispersion, as depicted in Table 2.

In order to further increase the channel utilization rate, in 2022, Shang et al. [108] proposed a multiplexed vortex state array consisting of multiple multiplexed vortex beams, which allows for flexible manipulation of both the spatial position and the corresponding OAM spectrum as desired (Figure 1c1). Then, employing an array as a data-carrier, the research group demonstrated a one-to-many multicasting link through multi-state OAM shift keying, where four various states were utilized to encode a four-bit symbol, achieving high-dimensional encoding; the experimental setup is shown in Figure 1c2. The Gaussian beam generated by a distributed feedback laser diode is converted into a horizontal linear polarization state by a half-wave plate (HWP) and a polarization beam splitter (PBS), and then the multicast OAM is encoded by a specially constructed phase-only liquid-crystal SLM. SLM1 is employed to carry out multicast OAM encoding. By switching holograms loaded on SLM1 continuously, a coded time-varying multiplexed vortex state array sequence is generated, which records the input signals. The array contains three diffraction orders. Regarding every diffraction order as a channel, there are three channels to send information, which are labeled as channel 1 to channel 3. After propagating 1.5 m in free space, the three encoded multiplexed vortex beams are received and decoded by three identical receivers consisting of an SLR camera, a lens, and an infrared CCD camera. In a subsequent demonstration, the research group generated different grayscale images from one transmitter to each of the three receivers. The results showed that the images received by each receiver were basically consistent with the transmitted signals, and the total bit error rate(BER) of the three channels was only 4.16 × 10^−4^, indicating successful multicasting of various signals, as presented in Table 2.

**Figure 2:**
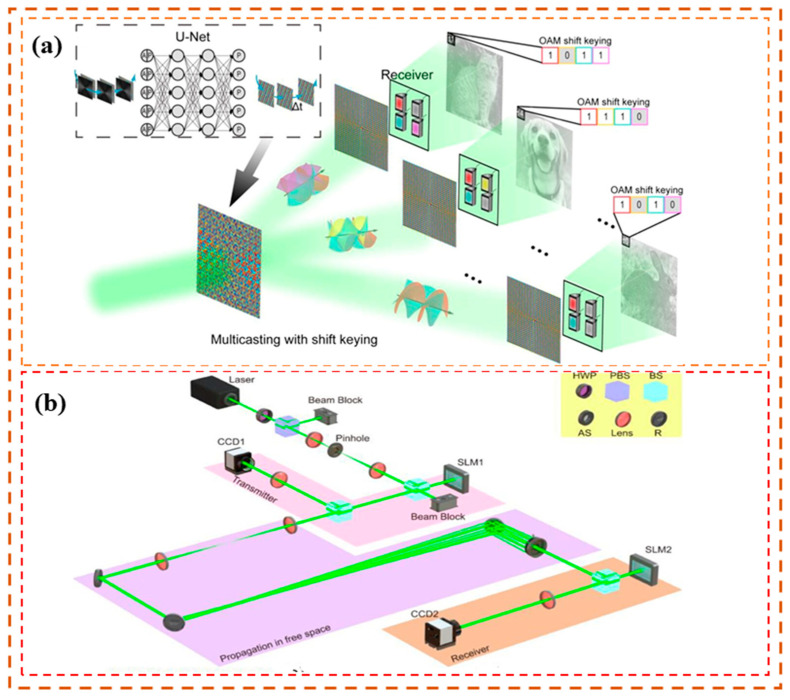
Working principle and experimental setup of OAM-RDs based on OAM channel multicasting (II) [109]: (**a**) principle of OAM neural communication, and (**b**) experimental setup.

Although the aforementioned methods achieved promising results, the overall complexity of the spatial frequency distribution increases considerably with an increase in the number of multiplexing channels. This adversely affects the hologram generation for multiplexed vortex beams, resulting in an increase in the OAM spectrum error. Therefore, a tradeoff is made between the bit error rate (BER) and the number of multiplexed channels. To address the above problem, in 2023, Meng et al. [109] reported a unique concept of OAM neural communication employing a network model based on a U-net neural network (Figure 2a) to transform the complex amplitude transmission functions into phase-only holograms (Figure 2b) [109]. The phase-only hologram predicted by their proposed network model generated a high-precision OAM spectrum for OAM multicasting with OAM-SK using only a few training datasets. At the transmitter, the information is sent to multiple users simultaneously when a beam of light passes through the hologram. Based on OAM-SK, the data at each user are encoded, and four different OAM states are selected to represent a 4-bit symbol, as can be seen in Figure 2a. Gray is used for a 0 weight OAM state, whereas OAM state 1 is represented by white. Decoding is performed at the receiver using a well-designed multiplexed OAM phase plate. A gray-scale algorithm measures the relative power of the four regions and translates it into the corresponding data. By switching holograms in the time sequence, real-time OAM optical communication can be achieved because, once trained, the network model can realize the noniterative calculation of all input signals. The hardware refresh time indicates the switching interval. To experimentally demonstrate the proposed concept, Meng and coworkers established 1-to-40 multicasting with 16-ary shift keying in OAM communication and achieved the encoding and decoding of 40 images with 16 gray values with a BER of 0 following a 3 m free space transmission.

Using the design of various OAM-RDs and related technologies, starting from the one-to-many multicast link in an ideal environment to the multicast link under diverse practical conditions, the OAM channel’s multicast technology has gradually matured. This is highly significant for reducing data congestion and enhancing efficiency and performance through multi-user data multicast. In addition, with the continuous development of hardware technology, the number of OAM multicasting channels has greatly increased, which is advantageous for its application in the network [110,111,112]. Thus, the OAM multicasting routers have become one of the important devices in today’s complex optical communication networks and have broad development prospects. Table 2 summarizes the representative works reported to date for the channel multicasting routers in OAM-based optical communication.

## 3. OAM Channel Switching

For the OAM optical communication system, it might be useful to extend the employment of an OAM-based communications link in a multi-user environment, in which different OAM data channels could be selectively manipulated. One of the OAM-RDs that can realize channel switching plays an important role [113,114,115,116,117,118,119]. In 2013, Yue et al. [120] experimentally demonstrated OAM-based reconfigurable optical switching functions when multiple OAM modes were used by switching *n* input modes with arbitrary OAM charges to *n* OAM modes with a desired number of charges. The concept of OAM-based reconfigurable optical networking functions can be described in parts “Charge shift”, “Charge exchange”, and “Charge selective manipulation”. “Charge shift” can realize the *m-l* shift of all OAM modes by using an SLM with topological charge *m-l* and a mirror. “Charge exchange” can reverse the order of OAM modes by using only one SLM, thus realizing the information exchange between the two OAM modes. “Charge selective manipulation” combines the functions of “Charge shift” and “Charge exchange” to easily manipulate the number of OAM modes using two SLMs without affecting the OAM of other modes. By effectively concatenating these three functions, it is possible to reposition any input of *N* OAM modes to any desired output state. Then, the team experimentally verified the feasibility of this OAM-based reconfigurable optical networking function system. In multi-pair OAM mode switching, the average conversion efficiency is −3.6 dB. At a BER of 2 × 10^−3^ (enhanced FEC (EFEC) threshold), the average OSNR penalties for the channels before and after exchanges are 1.5 dB and 2.4 dB, respectively. This research content has a very important influence on the OAM optical communication industry, and provides a new idea for the subsequent research in the design of router parts, as shown in Table 3

**Figure 3:**
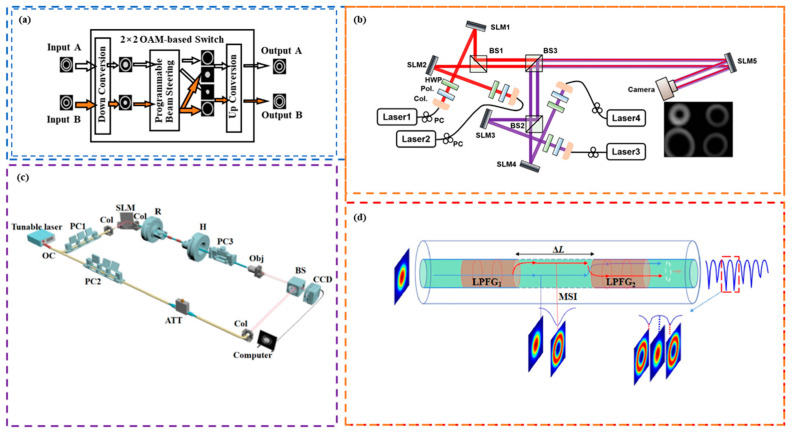
Principle or experimental setup of OAM-RDs based on OAM channel switching(I) (**a**) Functional block diagram of the 2 × 2 OAM-based switch [122]; (**b**) experimental setup of reconfigurable 4 × 4 OAM mode switching, space switching, and joint OAM mode and space switching [123]; (**c**) the schematic diagram of multi-channel MSI [118]; (**d**) experimental setup for the mode generation, dynamic switching, and detection between OAM modes. OC, 50:50 optical coupler; PC, polarization controller; H, holder; R, rotator; Obj, objective; ATT, attenuator; BS, beam splitter; Col, collimator; SLM, spatial light modulator; CCD, charge-coupled device [118].

Low-cost channel switching can also be realized by directing two composite vortex beams with different data and OAM mode values of *l*1 and *l*2 on a reflective SLM loaded with an OAM mode value of *l*R = −(*l*1 + *l*2). This converts the OAM mode values to −*l*2 and −*l*1 [121]. Taking the research further, Ahmed et al. [122] investigated a reconfigurable OAM channel switch with a 2 × 2 layout in 2013. The group experimentally demonstrated that multiplexed OAM beams can be selectively re-sent to different outputs by using multiple SLMs to first split the separated beams in space, followed by rerouting the separated beams. A schematic of the 2 × 2 OAM switcher that can either pass or redirect each input OAM beam can be seen in Figure 3a. Initially, the multiplexed input beam is converted into a Gaussian-like beam. At this time, the mode values of other OAM beams also change accordingly. After that, the OAM beam and the Gaussian beam are separated in space by employing a specially designed beam control phase mode, and the coaxial recombination of the Gaussian beam and OAM beam on another path is realized. Finally, the composite beam’s mode up-conversion is accomplished using the programmable SLM. In order to complete the mode up-conversion of the composite beam and the switching of the dual OAM channels, the center and peripheral phases of the composite beam correspond to distinct OAM modes. This allows the Gaussian beam to be restored to an appropriate OAM beam. The research group applied two multiplexed beams with OAM modes of *l* = +4, −4, and *l* = +2, −6, respectively, to input ports A and B. After system switching, the two multiplexed beams could complete the arbitrary switching of the OAM mode, and the signal-to-noise ratio was less than 2.5 dB, as presented in Table 3.

In addition to channel switching between the different OAM modes, the researchers then focused on spatial location switching. In 2016, Liu et al. [123] proposed a reconfigurable OAM mode-switching and spatial-switching scheme. By using multiple SLMs, this scheme realized the arbitrary control of the output position of the beam space while regulating the mode value of the input OAM beam. Thus, the tasks of OAM mode switching, space switching, joint OAM mode, and space switching can be completed in synch with each other. The experimental settings are shown in Figure 3b. Four channels transmit four different OAM beams generated by SLM1–SLM4 to specific locations in the spatial domain, which are then collected by three unpolarized beam splitters, BS1–BS3. BS1 collects the beams from SLM1 and SLM2 and splits them horizontally, whereas BS2 collects the beams from SLM3 and SLM4 and splits them horizontally. SLM5 emulates a 4 × 4 switching node. The four input ports of the switching node are linked to the four OAM channels after BS3, and the four output ports are projected onto a camera for monitoring. Thus, 4 × 4 OAM mode switching, space switching, and joint OAM mode and space switching are realized. This scheme further expands the utilization range of the optical networks and improves the flexibility of network control, as illustrated in Table 3. However, the use of multiple SLMs in the process increases the difficulty of loading phase designs on different SLMs and increases the system complexity and device cost to a certain extent. This has certain constraints on the high-speed optical communication link system, which requires high real-time and economic performance.

Very recently, Wu et al. [118] proposed a unique concept of multi-channel (OAM) mode switching based on an in-fiber mode selective interferometer (MSI) formed in a four-mode fiber. The MSI is made up of two strongly modulated long-period fiber gratings (LPFGs), which realize the mode switching between the selected mode pair (OAM_0_ and OAM_2_). By applying torsion to the MSI, the propagation constant of the two resonant modes will change, resulting in the control of the phase difference between both modes to switch at multiple channels. The experimental setup and the schematic diagram of the multi-channel MSI can be seen in Figure 3c and Figure 3d, respectively.

**Figure 4:**
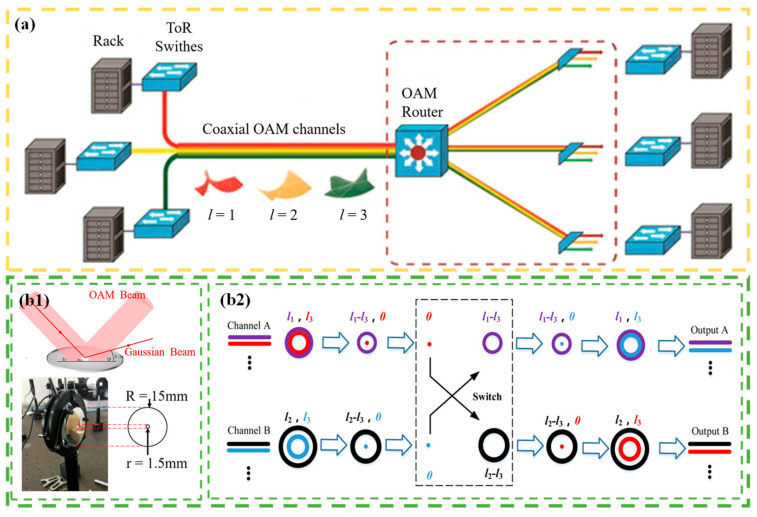
Principle or experimental setup of OAM-RDs based on OAM channel switching(II): (**a**) schematic of OAM-RD for intra-datacenter optical interconnects [124]; (**b1**,**b2**) the reconfigurable optical switching based on OAM [126]; (**b1**) dual-area mirror and principle of separating multiplexed beams; and (**b2**) principle of OAM-based optical switching.

The majority of the reports discussed above use SLM to realize the function of OAM routing. Although the OAM beam generated by SLM has advantages in terms of conversion efficiency and energy consumption reduction, the switching speed is limited by the material properties of the device itself. In 2017, Lei et al. [124] proposed an OAM-RD that used digital micromirrors instead of SLMs. They applied the device to an optical communication link system based on the data interconnection of OAM selection, as shown in Figure 4a. The optical router uses a binary grating driven by a digital micromirror to break the correspondence between the traditional OAM mode and diffraction order and can not only realize the mutual switching of input OAM modes but also ensure the function of multicast. In general, the scheme achieves the routing function of OAM channel switching well and also utilizes the advantage of a digital micromirror to greatly improve the switching speed, as shown in Table 3. In 2018, an OAM wavelength switch exploiting four OAM modes and four wavelengths was demonstrated by exploiting an integrated OAM multiplexer with four concentric waveguides, which allows for four input ports [125]. In 2021, Chen et al. [126] proposed an optical separation device composed of an SLM and a double-area mirror. They used On–Off Keying (OOK) to modulate four channels to form two inputs, channel A and channel B, each channel with two multiplexed OAM beams. An SLM was utilized to modify the OAM-multiplexed beams in each channel, with one of the beams being transformed into a Gaussian beam. The Gaussian beam was separated by a double-area mirror, thus enabling the switching of the beams carrying different data in the two channels. Figure 4b1 shows the principal architecture of a 2 × 2 OAM switcher. The double-area mirror is composed of two different mirrors. One was placed in the central region to reflect the Gaussian beam and the other in the outer region to reflect the OAM beam. Therefore, the separation of the Gaussian beam and OAM beam can be realized only by adjusting the angle between the inner and outer regions of the double-area mirror. As shown in Figure 4b2, two multiplexed beams with OAM modes of *l*_1_, *l*_3_s and *l*_2_, *l*_3_ were applied to input ports A and B, respectively. One beam at each input port was replaced with a Gaussian beam, and the other beam, still carrying the OAM state, passed through the double-area mirror for beam separation. The Gaussian beams extracted from channel A and channel B were introduced into the OAM beams of the other channels, creating mutual coupling. The reconstituted beam was coaxially transmitted and added at the corresponding phase to convert the multiplexed beams of channel A and channel B into the initial topological charge state, thus enabling the data interchange carried by the beam. The proposed method has the advantages of a simple structure, low cost, easy integration, and large angle separation, and can be applied to various OAM reconstruction scenarios, as depicted in Table 3. This facilitates a large-scale flexible OAM network construction.

Although the reported works above achieved promising results, a significant breakthrough in OAM switching was reported by Scaffardi et al. in 2023, reporting a 10 OAM × 16 wavelengths two-layer switch for 19.2 Tb/s data traffic using an integrated mode multiplexer [127]. They employed a 16-channel wavelength division multiplexing (WDM) grid, which enabled the switch to receive 160 optical Gaussian data inputs and route each input signal to a distinct output port, all of which take advantage of 10 OAMs. The foundation of this optical switch is built on an integrated OAM multiplexer and a tiny OAM demultiplexer. When verified experimentally, a 19.2 Tb/s total enabled throughput is made possible by each port’s 30 GHz bandwidth. The switching time is observed to be less than 1 μs. Since the OAM demux is passive, the OAM switch power consumption is 1.35 mW/Gb/s, which may be entirely attributed to the thermal tuning of the OAM emitters. This is advantageous compared to single-layer switches that cascade elementary blocks, such as 2 × 2, in order to achieve enormous port counts that increase in proportion to the square of the number of ports. The switch is compatible with standard telecom devices because it accepts input and output signals with Gaussian phase profiles that pass through optical fibers and waveguides.

At present, most OAM routers that can realize channel-switching functions switch the data information transmitted by different channels by controlling the change in the OAM beam mode value. In the past, the OAM switcher could switch data between two OAM beams, but with the continuous development of OAM multiplexing technology, the OAM switcher can now switch data between multiple composite beams [125,126,127]. With the development of OAM-RD, the flexible control of the OAM mode carried by multiplexed beams and the practicability of OAM communication links have been greatly improved, which lays a foundation for the research of OAM-RD. Table 3 summarizes the works reported to date for the channel-switching routers in OAM-based optical communication.

## 4. OAM Channel Filtering

**Figure 5:**
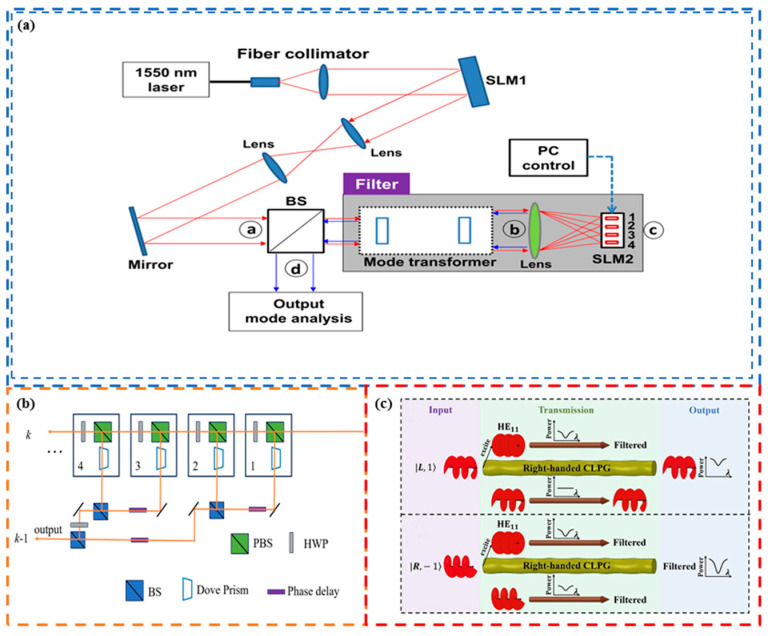
Principle or experimental setup of OAM-RDs based on OAM channel filtering: (**a**) the tunable mode-blocking filter [134]. (**b**) Schematic diagram of OAM filter based on multiple-beam interference [136]. (**c**) Schematic diagram of the transmission loss in the righthanded CLPG [137].

According to different applications, users have different requirements for channels where different OAM beams are located. When users have requirements for specific channels in multiplexed OAM communication links, it becomes increasingly urgent to study the filtering technology that filters out useless channels and only extracts specific channels according to the requirements [128,129,130,131,132,133]. Using the optical geometric transformation, Huang et al. [134] presented an OAM-RD in 2014 that could implement OAM filtering as can be seen in Figure 5a. The filtering operation works as follows: first, the light from the laser source is collimated and incident on SLM1, which has been pre-loaded with phase holograms designed to produce multiple superimposed OAM modes. The resulting OAM beam is sent into an OAM filter consisting of an SLM to control the light plate and a mode converter made up of two reflective elements used to convert phases. Based on the principle of optical geometric transformation, after the multiplexed OAM beam passes through the mode converter, the original annular distribution of light intensity is converted into a series of strip light spots whose distribution corresponds to the OAM mode value one by one. The light spot is then brought into focus on the focal plane using a convex lens. At various positions, the light spot can be selectively controlled using the SLM. The spot of OAM beam filtering required by the target is blocked and reflected at the same time. Finally, based on the principle of optical geometric inversion, the reflected light spots are recovered from the light spots to the normal annular OAM beam, and the beam splitter is used to separate them. Thus, the filtering operation of the required OAM beam components in the composite OAM beam is completed. The group experimentally demonstrated that the blocking mode and propagation mode output power suppression ratios are greater than 14.5 dB. However, when the mode value interval of the input composite OAM beams is 1, the light spots will overlap following geometric transformation, which results in considerable difficulty in subsequent filtering. This is because of the inherent limitations of optical geometry transformation.

In order to further optimize the OAM multicast filter element and change the fixed relationship between diffraction order and OAM mode in the traditional computational hologram, in 2016, Gao et al. [135] proposed a new routing scheme based on OAM-labeled. First, several Gaussian beams are incident at specific incidence angles to reach the Dammann optical vortex grating (DOVX), so as to ensure multiple coaxial OAM beams of different OAM beams are generated at the outgoing end. The receiver incident the OAM beam into a specially designed computer-generated holograph (CGH). By taking the relative root-mean-square error as the evaluation factor of the hologram, the pure phase transmittance function is optimized using the iterative method, and the phase distribution of the CGH is dynamically controlled. Multiple OAM beams are reconverted into Gaussian-like beams by using a CGH, which can then be routed to different output ports. These OAM-labeled beams can be manipulated through direct connection, switching, multicast, and filtering functionalities. These routing functions can be realized by reconstructing CGH. The team verified the OAM filtering in the subsequent experiments, and the experimental results are highly consistent with the theoretical results, and the blocked output port power is suppressed by more than 23 dB by the filter, as shown in Table 4.

In order to realize the high-fidelity output of OAM mode, in 2019, Li et al. [136] combined the advantages of the interferometer and proposed another novel OAM filter using the interferometer. In this scheme, multi-beam interference filtering is realized by controlling the relative phase difference in the different OAM beams. The principle is shown in Figure 5b. First, the polarized beam splitter (PBS) is used to divide the input *n* OAM multiplexed beams into *n* equal parts, and then the relative phase difference in the different OAM beams is controlled by the Dow prism. Additionally, a half-wave plate (HWP) is utilized to counteract the half-wave loss due to the reflection at each branch interface. Moreover, a series of phase compensators are placed to adjust for the phase discrepancies arising from the different optical path branches. Since the relationship between OAM mode and phase difference satisfies *θ* = 2π*l*, only a specific OAM mode value can achieve 100% passing, while other OAM modes will be suppressed, so as to realize the OAM filtering function as presented in Table 4. This filtering reduces the overlapping effect of adjacent OAM modes and improves the filtering effect. It can be used in the fields of single photon recognition and optical switching.

In 2022, Rao et al. [137] proposed and experimentally demonstrated a chiral long-period fiber grating (CLPG)-based scheme for filtering spin-entangled OAM of photons by utilizing the inherent spiral characteristics of a CLPG. The authors demonstrated both theoretically and experimentally that co-handed OAM, characterized by the same chirality as the helical phase wavefront of a CLPG, couples with higher-order cladding modes and experiences a loss. Conversely, cross-handed OAM with the opposite chirality propagates freely through the CLPG. To input |***L***, 1〉 and |***R***, −1〉, for example, on the right side of CLPG, a transmission loss is observed, as shown in Figure 5c. Since the spatial light coupling at the input end to the fiber will inevitably excite the fundamental mode (***HE***11), there will be not only the input vector vortex beam (VVB) but also the ***HE***11 mode in the grating. In addition, the excited ***HE***11 mode will be coupled with the cladding ***HE***21 mode, and the loss will occur near the resonant wavelength. The ***HE***11 modes excited by input |***L***, 1〉 and |***R***, −1〉 are the same. Excluding the loss from ***HE***11 coupling, there will be no extra loss as the input VVB |***L***, 1〉 will freely pass, while the input VVB |***R***, −1〉 will couple to the cladding mode and suffer loss. Therefore, VVB |***R***, −1〉 will suffer more loss than VVB |***L***, 1〉 and their difference is the loss caused by chirality selected VVB |***R***, −1〉 coupling. Thus, the filtering function is realized. In contrast to an OAM filter based on twisted photonic crystal fiber, the CLPG-based scheme has the advantages of narrow bandwidth and wavelength selectivity. In contrast to the OAM filters based on spatial optical devices, the CLPG-based scheme has the advantages of small size and good compatibility with the existing fiber-based OAM applications, which is promising for all-fiber OAM multiplexing communications, as illustrated in Table 4.

The OAM filter routers can filter out unnecessary OAM beams via the selective filtering of multiplexed OAM beams, which meets the requirement of channel filtering of specific nodes in optical networks [138,139,140]. Initially, the OAM filter components only filtered OAM beams with large mode spacing. However, it is now possible to carry out high-fidelity filtering for any OAM beam, which has a broad application prospect in future optical communication networks. Table 4 summarizes the works reported to date for the channel filtering OAM-based optical communication routers.

## 5. OAM Channel Hopping

**Figure 6:**
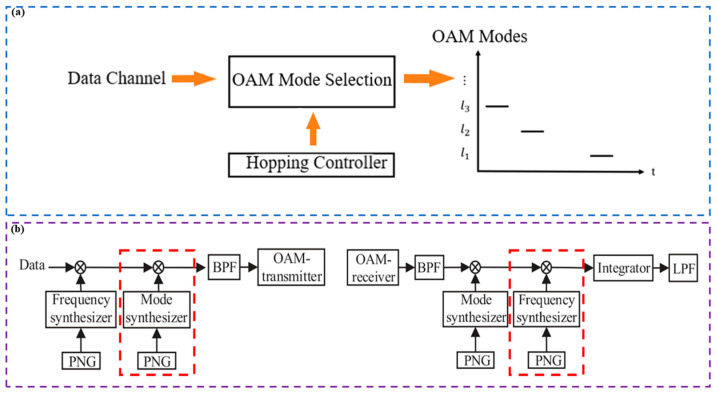
Principle or experimental setup of OAM-RDs based on OAM channel hopping: (**a**) spatial domain channel hopping principle based on OAM mode [145]; (**b**) the schematic diagram MFH system model [146].

There are various types of OAM-RD, and in addition to the numerous categories mentioned above, a device capable of channel hopping has gradually come into view [141,142,143,144]. This device can be used in a reconfigurable multiple-access network to output different OAM modes at different time windows. Willner and coworkers proposed the channel-hopping OAM-RD in 2015 [145]. Its working is similar to frequency hopping in conventional transmissions and can be realized by controlling the hop mode controller to output the desired OAM mode for a specific time from a particular output port (Figure 6a). In this scheme, the use of a digital micromirror instead of an SLM effectively improves the speed of channel hopping and can be used to arbitrarily route data streams based on the spatial pattern occupied by the channel in a multi-access optical network. Willner and group succeeded in performing reconfigurable hopping of a QPSK data channel at a rate of 100 Gbit/s (with a switching protection time of 2 ns), with a signal carrying four OAM modes keeping the power loss below 5.3 dB.

In order to achieve efficient channel hopping performance of OAM communication in 2018, Liang et al. [146] combined the mode hopping (MH) scheme with the traditional frequency modulation scheme and proposed a mode frequency hopping (MFH) scheme. The system model is shown in Figure 6b (the transmitter on the left and the receiver on the right). The MFH system is based on the MH system by adding two frequency synthesizers (as shown in the red dotted line in Figure 6b). The OAM transmitter and OAM receiver can utilize a uniform circular array (UCA) antenna configuration. In the case of the OAM transmitter, the N array elements are fed with the same input signal, with each successive element receiving a progressively delayed signal so that the phase of the wavefront is incremented by an integer multiple l of 2π after a complete turn. The input data symbol experiences U OAM mode or frequency hops. At the transmitter, a mode/frequency synthesizer, controlled by a pseudorandom noise sequence generator (PNG), selects an OAM mode or a frequency band range. To de-hop the OAM mode at the receiver, PNGs identical to those used in the transmitter are employed. The received signal is processed through an integrator and low-pass filter to recover the transmitted signal. The research group then compared the BERs of the scheme it developed with traditional wideband frequency modulation schemes. The numerical results show that the communication BER of the MFH scheme developed by the research group is much lower than that of the traditional wireless frequency modulation scheme, which can improve the anti-interference performance of wireless communication systems effectively, as illustrated in Table 5.

In 2022, Liang et al. introduced an index-modulation embedded mode-hopping (IM-MH) scheme [147]. This innovative scheme enables the simultaneous activation of multiple OAM modes for hopping, while also facilitating the transmission of additional index information and signal data. An analysis of the average bit error rates (ABERs) for the proposed IM-MH scheme under two scenarios—perfect channel state information (CSI) and imperfect CSI—was performed. Additionally, they propose the index-modulation embedded double-serial MH (IM-DSMH) scheme, which incorporates a random activation of one OAM mode as the second serial hop in transmitting the hopping signals within the IM-MH scheme. This further reduces the average bit error rates (ABER) in wireless communications. The input signal information is divided into signal and index components. By utilizing secure keys to determine the index information, index selector A selectively activates I out of N OAM modes, enabling the realization of OAM multiplexing at each hop. The inactive state is assigned to the remaining (N–I) OAM modes. During the operation of the Double-Serial Mode-Hopping (DSMH), index selector B selectively activates one OAM mode as the second serial hop based on the index information. The activation pattern of OAM modes follows a consistent sequence for both transmitters and receivers while appearing random to potential attackers. Extensive numerical analysis demonstrates the effectiveness of their proposed schemes in achieving low Average Bit Error Rates (ABER) and significantly increasing Spectral Efficiency (SE) within a narrowband. Specifically, the ABERs of the proposed IM-MH and IM-DSMH schemes are approximately 25% and 10% of the traditional schemes, respectively.

At present, most OAM-RD which can realize the channel switching function realizes the strong anti-interference ability of wireless communication by mode hopping or frequency hopping [148,149]. However, only one OAM mode/band is excited to load and transmit line information per hop, resulting in low-capacity problems caused by low OAM mode or spectral interest rate. This problem is at odds with the increasing data rate requirements of wireless communications. Therefore, how to improve the OAM-RD which can realize channel hopping is a priority in future research direction. Table 5 summarizes the works reported to date for the channel hopping routers OAM-based optical communication.

## 6. OAM Channel Adding/Extracting

**Figure 7:**
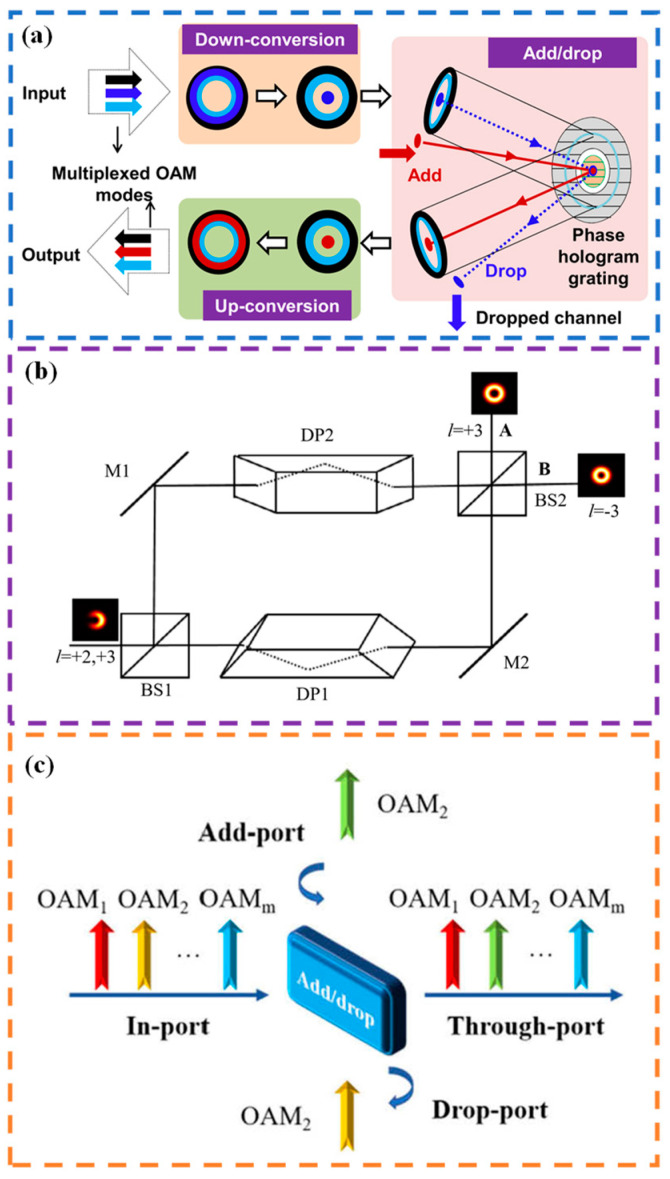
Principle or experimental setup of OAM-RD for OAM channel adding/extracting: (**a**) concept of OAM channel add/drop multiplexing [152]; (**b**) the schematic of the modified MZI [154], BS: beam splitter, M: mirror, and DP: Dove prism; (**c**) concept of OADM for OAM add-drop multiplexing [155].

Selective channel adding or extracting is also an important part of optical communication links [150,151]. In 2013, Huang et al. [152] reported an OAM mode division multiplexer that efficiently added or extracted a single OAM beam. Figure 7a gives the working mechanism of the proposed experimental setup. The desired OAM beam is transformed into a Gaussian beam while the mode values of other OAM beams remain unchanged. Afterward, using an SLM loaded with two different region grating phases, a composite beam is separated. To filter out the center Gaussian beam of the coaxial composite beam, the inner grating changes the diffraction direction of the Gaussian beam, while the outer grating does not change the diffraction direction of the OAM beam, enabling the OAM beam to reflect along its normal path and thus extract the target OAM beam channel. For a 100 Gbit/s QPSK data channel, this scheme incurs a power loss of less than 2 dB, and the BER of the channel is only 2.0 × 10^−3^, as can be seen in Table 6. Although this OAM-RD can flexibly manipulate a single OAM channel, controlling the extraction or addition of multiple OAM channels is still a challenge due to the power loss caused by crosstalk between channels.

**Table 6:** Summary of channel adding/extracting routers used for the OAM-based optical communications.

Year	Methods	Equipment	Strengths/ Weaknesses	Transmission Result	Ref.
2013	Reconfigurable multiplexers	SLM	Operate channels flexibly/Only one channel at a time	The power loss of this scheme is less than 2 dB and the channel error rate is only 2.0 × 10^−3^.	[152]
2019	Geometric transformation	SLM	Realize the addition and extraction/Required multiple SLMs	The energy purity of the measured OAM is about 95%.	[153]
2019	OAM analyzer	Interferometer, Dove prisms	Separate the odd and even OAM beams/-	The crosstalk values are below −10 dB and can sort the odd and even OAM beams	[154]
2021	OAM add-drop multiplexer	diffractive deep neural network	Diffraction efficiency and mode purity are very high/ -	Signal-to-noise ratio penalties of ~1 dB at a BER of 3.8 × 10^−3^	[155]

To add or extract multiple OAM channels, in 2019, Feng et al. [153] developed a geometric transformation-based OAM mode division multiplexer that successfully realized the extraction and addition of multiple OAM channels. First, a series of narrow light spots corresponding to the OAM mode values are obtained by using two SLMs loaded with special phases and a Fourier lens to transform the incoming OAM multiplexed beam based on the principle of optical geometric transformation. Then, the target OAM beam to be extracted is transmitted by setting the reflection phase on the SLM, while the other OAM beams remain reflected. Finally, the reflected light path passes through the previous optical components to complete the optical geometric inverse transformation and restore the original OAM beam, thereby achieving the extraction of multiple OAM beams in the composite beam. When it is necessary to add multiple OAM beams, the desired OAM beams to be added are first transformed into narrow light spots at the focal plane position through optical geometric transformation. Then, the SLM is adjusted to transmit the target OAM beam to be added while reflecting other OAM beams. After the reverse transmission along the same optical path, the added beam and the original beam are restored into a new OAM multiplexed beam at the output end through a beam splitter and optical geometric inverse transformation, thus completing the OAM beam adding operation. This scheme can not only realize the adding and extracting of OAM beams simultaneously but also further duplicate the other OAM modes in the composite OAM beam without affecting them, reducing the spot size to maintain the high energy purity of the OAM beam, as depicted in Table 6.

In 2019, Feng et al. [154] introduced a high-density OAM analyzer comprising two OAM mode converters and a modified Mach–Zehnder interferometer (MZI). This MZI, which includes a pair of Dove prisms in addition to the standardized MZI configuration, effectively avoids the inherent overlap of adjacent OAM modes within the mode converter. In this scheme, the multiplexing OAM beam is incident into the modified MZI, which can sort and separate the even and odd OAM modes into different ports, respectively. The schematic of the modified MZI is shown in Figure 7b: when the input beam is incident to the beam splitter of BS1, the beam is divided into two beams. In the upper path, the reflected beam is first reflected by M1 and then passes through DP2, after which it is split into two beams by BS2. One beam reaches port A of BS2 while the other is reflected towards port B. Conversely, in the lower path, the transmitted beam first passes through DP1 and is then reflected by M2 before being split by BS2. One of the resulting beams is directed towards port A of the BS2, while the other reaches port B. The proposed method ensures that the topological charge interval between adjacent OAM beams is at least equal to 2 at both output ports of the MZI. As a result, the distance between the converted light spots corresponding to these OAM modes will be greater than the width of each individual spot, effectively avoiding any overlapping and enabling high-density OAM mode analysis with enhanced discrimination resolution, as presented in Table 6. This feature addresses a key challenge in the OAM mode analysis, where previously the ability to analyze high-density OAM modes was effectively constrained. 

Generally, phase gratings are employed in the construction of the OAM add–drop multiplexer (OADM). However, the rigid construction and beam-splitting properties of phase gratings restrict their diffraction efficiency, adaptability, and usefulness. In recent years, deep neural networks have emerged as a promising technique for information processing in the OAM mode. These networks have the capacity to theoretically approximate functional relationships in any input–output domain. In 2021, Xiong et al. [155] introduced an OAM add–drop multiplexer (OADM) utilizing an optical diffractive deep neural network (ODNN). By leveraging the powerful data-fitting capability of deep neural networks and the intricate light-field manipulation abilities of multilayer diffraction screens, a five-layer ODNN was constructed. This ODNN enables precise control over the spatial positioning of vortex beams, facilitating the selective coupling and separation of OAM modes. The specific structure of the OAM add-drop multiplexer (OADM) using the optical diffractive deep neural network (ODNN) is depicted in Figure 7c. The input plane of the diffraction neural network is divided into several ports: (1) in port: this port receives the multiplexed OAM channels; (2) drop port: it extracts the selected OAM channel from the multiplexed channels; (3) add port: this port uploads an OAM channel carrying a new data stream; (4) through port: it outputs the re-multiplexed OAM channels. By leveraging the add and drop functions, the OADM can selectively upload and download OAM channels without affecting other transmitted OAM channels. In simulations, both the diffraction efficiency and mode purity exceeded 95%. Four OAM channels carrying 16-quadrature amplitude modulation signals were successfully downloaded and uploaded with only 1 dB loss in optical signal-to-noise ratio at a BER of 3.8 × 10^−3^. This innovative approach overcomes the limitations of conventional OADMs, which have single functionalities and poor flexibility. It provides new possibilities for OAM multiplexing and all-optical interconnection.

In general, the research on the OAM-RD that can implement channel addition/extraction is still in its infancy, and more in-depth related research is needed. These studies have developed from the extraction and addition of a single OAM beam to the extraction and addition of multiple OAM beams, which will improve the expansion ability of OAM channels step by step, and build the foundation for realizing multi-channel networks, in which intermediate-node users can access their selected channels without interfering with other channels. Table 6 summarizes the works reported to date for the channel adding/extracting routers OAM-based optical communication.

## 7. Conclusions

This paper mainly reviews and summarizes the research achievements of OAM-RDs for OAM-based optical communication. According to different router functions, the router components can be divided into five categories, including OAM channel multicasting, switching, filtering, hopping, and adding/extracting. This review briefly introduced the working principle and development history of different OAM-RDs.

With further development of the OAM-based optical communication technology, OAM-RDs play important roles in improving the flexibility and practicability of optical communication systems. OAM multicasting devices can copy the data information of a single channel to multiple channels for better adaptation to the multi-user environment. OAM switching devices can switch the OAM mode value of the input beam to realize effective data switching between different OAM channels. OAM filtering devices can realize the extraction of specific channel information from the multiplexed OAM channels. OAM hopping devices, by changing the OAM modes or frequencies, can achieve channel switching to improve the anti-jamming ability of OAM-based optical communication. Meanwhile, OAM adding/extracting devices, employing the selective addition or extraction of the OAM communication channel, can access the selected channel without interfering with other channels for the intermediate-node users. So far, OAM-RDs are developing towards miniaturization and integration, which coincides with the requirements of devices in today’s optical communication. With in-depth research, the developments on the OAM-RDs have achieved a series of important breakthrough results, which play important roles in the OAM-based optical communication system.

OAM channel multicasting holds great potential to meet the increasing bandwidth demands in future optical communication networks as it allows a single optical signal to send data through a shared optical network to multiple receivers. However, significant efforts are still needed to send data to ultra-multi-users simultaneously without compromising the bit error rate (BER) of data transmission. Similarly, realizing low-cost channel switching with ultra-fast switching time is a challenge yet to be overcome. The use of multiple SLMs in the switching process increases the difficulty of loading phase design on different SLMs and increases the system complexity and device cost, putting constraints on the high-speed optical communication link system which requires high real-time and economic performance. OAM channel filtering router devices with small sizes and good compatibility with the existing fiber-based OAM applications are one of the future research directions. To achieve efficient channel hopping, the performance of combinational OAM communication schemes should be researched and studied to combine the benefits of both simultaneously. For efficient, reliable, and easily integrable OAM add–drop multiplexer (OADM), deep neural networks must be explored as the traditional rigid construction and beam splitting properties of phase gratings restrict their diffraction efficiency, adaptability, and usefulness. 

In summary, as a new optical communication technology, OAM-RDs for OAM-based optical communication have been widely explored by researchers, but there are still some challenges to overcome, which are attracting further research. In addition, OAM has developed in the field of optical communication, and it is also a broad application prospect in the field of acoustic communication and traditional RF communication. However, the OAM-RD research in the field of acoustic communication and traditional RF communication is still in its infancy. It is believed that with the continuous studies conducted by worldwide researchers, in the near future, there will be more functions and more optimized and flexible OAM-RDs in various fields to gradually promote the continuous development of wireless communication based on the OAM.

## Figures and Tables

**Table 1:** Schematic diagram of OAM router function and previous works related to OAM-RD.

OAM-RD	Schematic Diagram	Function	Reference
Multicasting	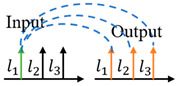	Extended communication link	[95,96,97,98,99,100,101,102,103,104,105,106,107,108,109,110,111,112]
Switching	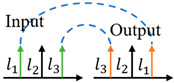	Exchanging data	[113,114,115,116,117,118,119,120,121,122,123,124,125,126,127]
Filtering	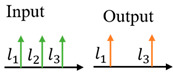	Extracting specific channel	[128,129,130,131,132,133,134,135,136,137,138,139,140]
Hopping	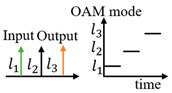	Anti-interference	[141,142,143,144,145,146,147,148,149]
Adding/ Extracting	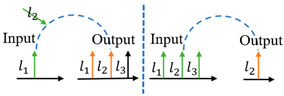	Improved the scalability of channels	[150,151,152,153,154,155]

**Table 2:** Summary of channel multicasting routers used for the OAM-based optical communications.

Year	Method	Input	Output	Equipment	Strengths/ Weaknesses	Transmission Result	Ref.
2013	Sliced phase patterns	Single OAM	Multiple same-mode-spacing OAMs	SLM	Low loss efficiency/ Low integration	Channel crosstalk < −20 dB	[105]
2015	Adaptive correction	Gaussian beam	Multiple collinear superposition OAM	SLM	Output power adjustable/ Limited mode selection	Maximum power deviation is ~0.3 dB	[106]
2022	Optical tapped-delay-lines	Gaussian beam	Multiple adjustable OAM	SLM	Reduced error vector magnitude (EVM)	Error-vector magnitude is ~12.9%	[107]
2022	Multiplexed vortex state array	Multiplex OAM	Multiple multiplexed OAM	SLM	High channel utilization/-	The mean square error is 1.06 × 10^−3^	[108]
2023	16-ary shift keying	Multiplex OAM	Multiple multiplexed OAM	SLM	-/Ultra-high precision, 1-to-40 multicasting	The mean square error is 0.02, BER of 0	[109]

**Table 3:** Summary of channel-switching routers used for the OAM-based optical communications.

Year	Methods	Equipment	Strengths/ Weaknesses	Transmission Result	Ref.
2013	Reconfigurable optical networking functions	SLM	Multi-pair data channel exchange/Cumbersome structure	The average conversion efficiency is −3.6 dB	[120]
2013	Mode overlay	SLM	Simple structure/ Only switch the single OAM	When the BER is 1 × 10^−9^, the power loss is less than 2.4 dB	[121]
2013	Reconfigurable 2 × 2 switch	SLM	-	Signal-to-noise ratio less than 2.5 dB	[122]
2016	Space switching	SLM	Adjustable output position/ Complex system and high device cost	-	[123]
2017	Optical vortex grating	Digital micromirror	Fast switching speed/-	The router has a fast-switching time of 6.9 μs	[124]
2021	Optical separation	Reflector and SLM	Simple structure and low cost/ -	-	[126]

**Table 4:** Summary of channel filtering routers used for the OAM-based optical communications.

Year	Methods	Equipment	Strengths/ Weaknesses	Transmission Result	Ref.
2014	Optical geometric transformation	SLM	Mode adjustable/ Difficult to filter out OAM with small mode intervals	The output power rejection ratio of blocking mode and propagation mode exceeds 14.5 dB.	[134]
2016	Labeling overlapped optical flows	Dammann optical vortex grating	Diversified function/-	The power of the blocked output port is suppressed by more than 23 dB by the filter	[135]
2019	Controlling the phase difference	Interferometer	Reduce the overlapping effect of adjacent OAM models/-	Specific OAM schema values can achieve 100% passing theoretically	[136]
2022	Chiral long-period fiber grating	SLM, grating	Without exerting extra loss for other OAM/-	-	[137]

**Table 5:** Summary of channel hopping routers used for the OAM-based optical communications (PNG, pseudorandom noise sequence generator; BPF, band pass filter; LPF, low pass filter; UCA, uniform circular array).

Year	Methods	Equipment	Hopping Type	Strengths/ Weaknesses	Transmission Result	Ref.
2015	Mode hopping controller	Digital micromirror	OAM mode	Fast Channel hopping/Additional losses	The measured power penalties are below 5.3 dB.	[145]
2018	Orthogonality of OAM modes	PNG, BPF, LPF, integrator	OAM mode, frequency	Low BER/ Low integration	The BER is much lower than the traditional method	[146]
2022	IM-MH scheme	Signal modulator, index selector, UCA	mode hopping	Low ABERs and Increase the SE/ -	The ABER is around 25% of the traditional scheme	[147]

## Data Availability

Not applicable.
